# Longitudinal observations of progressive cardiac dysfunction in a cardiomyopathic animal model by self-gated cine imaging based on 11.7-T magnetic resonance imaging

**DOI:** 10.1038/s41598-017-09755-4

**Published:** 2017-08-22

**Authors:** Shigeyoshi Saito, Minori Tanoue, Kasumi Masuda, Yuki Mori, Satoshi Nakatani, Yoshichika Yoshioka, Kenya Murase

**Affiliations:** 1 Department of Medical Physics and Engineering, Division of Health Sciences, Osaka University Graduate School of Medicine, Suita Osaka, 560-0871 Japan; 2 Center for Information and Neural Networks (CiNet), National Institute of Information and Communications Technology, Osaka University, Suita Osaka, 560-0871 Japan; 3 Department of Functional Diagnostics, Division of Health Sciences, Osaka University Graduate School of Medicine, Suita Osaka, 560-0871 Japan; 4 Biofunctional Imaging Laboratory, Immunology Frontier Research Center (IFReC), Osaka University, Suita Osaka, 560-0871 Japan

## Abstract

The purpose of this study was to longitudinally assess left ventricular function and wall thickness in a hamster model of cardiomyopathy using 11.7-T magnetic resonance imaging (MRI). MRI were performed for six cardiomyopathic J2N-k hamsters and six J2N-n hamsters at 5, 10, 15, and 20 weeks of age. Echocardiography was also performed at 20 weeks. The ejection fraction (EF) at 15 and 20 weeks of age in J2N-k hamsters showed a significant decrease compared with those in controls. Conversely, the end-systolic and end-diastolic volumes in cardiomyopathic hamsters showed a significant increase compared with those in controls. Moreover, the heart walls of J2N-k hamsters at 15 and 20 weeks were thicker than those of controls at end-systole; however, there were no significant differences at end-diastole. Optical microscopy with Masson’s trichrome staining depicted no fibrosis in the control myocardium, although it showed interstitial fibrosis in the 20-week-old J2N-k cardiomyopathic myocardium. There were no differences in EF and the wall thickness observed on MRI and those observed on echocardiography. These results indicate the presence of systolic dysfunction in cardiomyopathic hamsters. Self-gated cine imaging based on 11.7-T MRI can be used for serial measurements of cardiac function and wall thickness in a cardiomyopathic model.

## Introduction

Cardiomyopathy is a progressive disease of the myocardium or heart muscle. The major types of cardiomyopathy include dilated, hypertrophic, and restrictive cardiomyopathy. Dilated cardiomyopathy (DCM) is characterized by chamber dilation and impaired cardiac pump function. Although DCM is known to result in cardiac contractile dysfunction, the underlying mechanisms are unclear. The J2N-k hamster is a model used for studying cardiomyopathy. It exhibits a deletion in the δ-sarcoglycan (SG) gene expressed in striated muscles^1^. These hamsters show cardiomyopathy with eventual progression to heart failure and offer an appropriately matched healthy control, which is the J2N-n hamster. A previous study reported that an alteration in the sarcoplasmic reticulum function and its regulation contributed to cardiac contractile dysfunction in J2N-k cardiomyopathic hamsters^2^. Furthermore, Mitsuhashi *et al*.^3^ reported that these animals die from congestive heart failure at 1 year of age, while other studies performed at 36 weeks of age showed that these hamsters did not develop congestive heart failure^2^. Conversely, Mitsuhashi *et al*.^3^ have proposed that cardiac dilatation and dysfunction begin at 20 weeks in J2N-k hamsters. Therefore, it is important to clarify the timing of cardiac dysfunction in these cardiomyopathic hamsters. This will not only provide information on disease progression in a cardiomyopathic model but also aid in monitoring DCM treatment strategies for preclinical research.

In small animals, high-resolution electrocardiography (ECG) and respiratory-gated cine cardiac high-field magnetic resonance imaging (MRI) have been used to quantify cardiac function^4–7^. This cine MRI protocol, which is similar to human cardiac MRI, generates a stack of two-dimensional slices with a thickness of <1.0 mm along the main cardiac axis to achieve full cardiac coverage. Subsequently, ventricular volume segmentation at the end-diastole and end-systole is used to evaluate cardiac function. Recent approaches for left ventricular (LV) measurement are based on self-gated MR sequences in the absence of ECG and respiratory monitoring^8, 9^. These imaging sequences involve prospectively and retrospectively triggered (self-gated) protocols that are both useful and robust for the evaluation of mouse disease models using cardiac imaging^10–12^. In addition, these techniques can be used for serial cardiac function measurements in small animal models.

The purpose of this study was to longitudinally observe LV function and wall thickness in cardiomyopathic hamsters using a self-gated magnetic resonance (MR) sequence for ultra-high-field 11.7-T MR cine imaging.

## Results

### Basic characteristics and typical images of J2N-k and J2N-n hamsters

**Figure 1:**
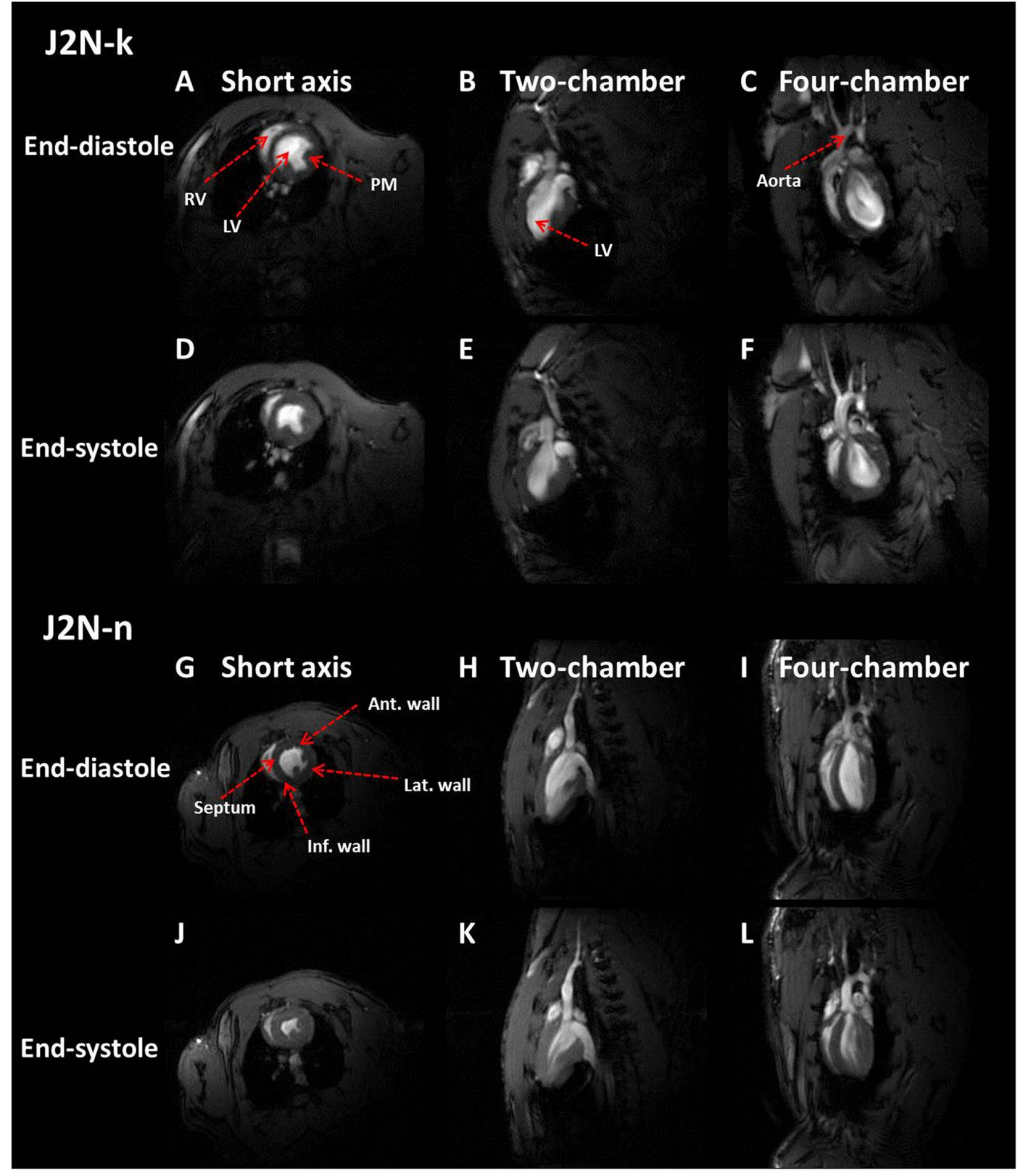
Examples of typical three-plane images of J2N-k cardiomyopathic and J2N-n control hamsters obtained in three different views. (**A**,**D**,**G** and **J**) Short-axis view. (**B**,**E**,**H** and **K**) Two-chamber view. (**C**,**F**,**I**, and **L**) Four-chamber view. The red arrows indicate the left ventricle (LV), right ventricle (RV), aorta, papillary muscles (PMs), anterior (Ant) wall, inferior (Inf) wall, septum, and the lateral (Lat) wall.

As shown in Table 1, the average body weight of J2N-k hamsters was comparable to that of J2N-n hamsters, even at 20 weeks of age. However, the average heart rate of J2N-k hamsters was lower than that of J2N-n hamsters at 20 weeks of age. Typical three-plane images of J2N-k cardiomyopathic and J2N-n control hamsters are shown in Fig. 1. Cardiac movies of the 10 phases in a single cardiac cycle are shown in Sup.1 (J2N-k cardiomyopathic hamster, four-chamber view) and Sup.2 (J2N-n control hamster, four-chamber). The heartbeat during one cardiac cycle can be observed using these movies.

**Table 1:** Basic characteristics of J2N-k cardiomyopathic and J2N-n control hamsters.

	Body weight (g)			Heart rate (bpm)		
	J2N-k	J2N-n	p	J2N-k	J2N-n	p
5-weeks	70.8 ± 6.7	71.3 ± 2.9	ns	404.3 ± 7.9	400.7 ± 3.1	ns
10-weeks	90.0 ± 6.8	95.7 ± 2.9	ns	395.8 ± 8.0	396.7 ± 8.2	ns
15-weeks	99.3 ± 1.0	102.2 ± 4.1	ns	400.2 ± 17.9	408.8 ± 14.8	ns
20-weeks	113.0 ± 3.5	107.7 ± 6.1	ns	378.3 ± 9.4	402.5 ± 9.1	<0.01

Values are expressed as means ± standard deviation.

### LV function of J2N-k cardiomyopathic and J2N-n control hamsters

**Figure 2:**
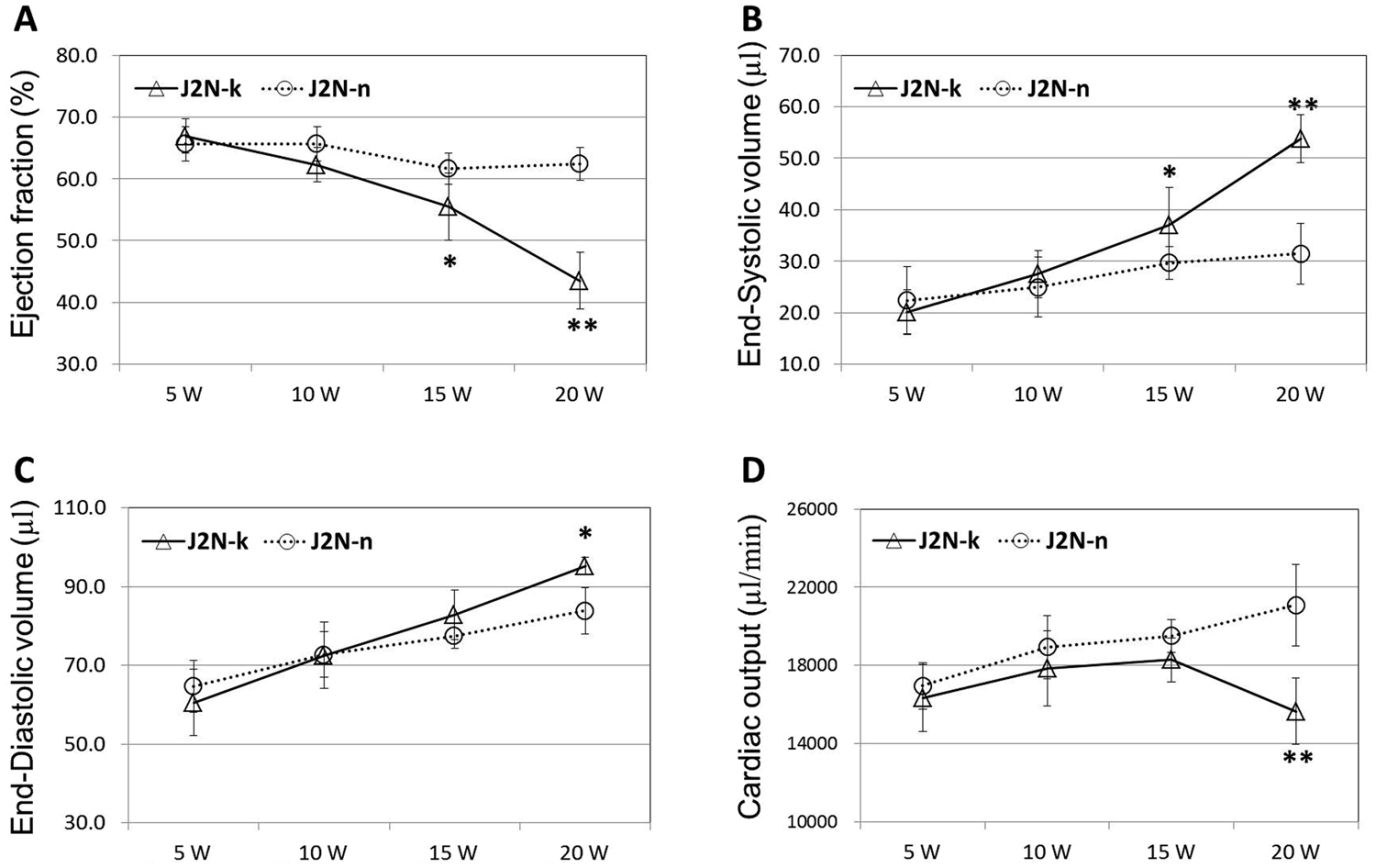
Left ventricular function in J2N-k cardiomyopathic and J2N-n control hamsters. A significant decrease in ejection fraction and cardiac output is observed in J2N-k hamsters at 15 and 20 weeks of age. However, a significant increase in end-systolic and end-diastolic volumes is observed in cardiomyopathic hamsters. *p < 0.05. *p < 0.01.

EF in J2N-k hamsters showed a significant decrease compared with that in controls at 15 (p < 0.05) and 20 weeks of age (p < 0.01, Fig. 2A). However, ESV in cardiomyopathic hamsters showed a significant increase compared with that in controls at 15 (p < 0.05) and 20 weeks of age (p < 0.01, Fig. 2B). As shown in Fig. 2C, the EDV in cardiomyopathic hamsters showed a significant increase compared with that in controls at 20 weeks of age (p < 0.05). Finally, the CO in J2N-k hamsters showed a significant decrease compared with that in controls at 20 weeks of age (p < 0.01, Fig. 2D).

### 3D color-coded mapping of LV wall thickness in J2N-k cardiomyopathic and J2N-n control hamsters

**Figure 3:**
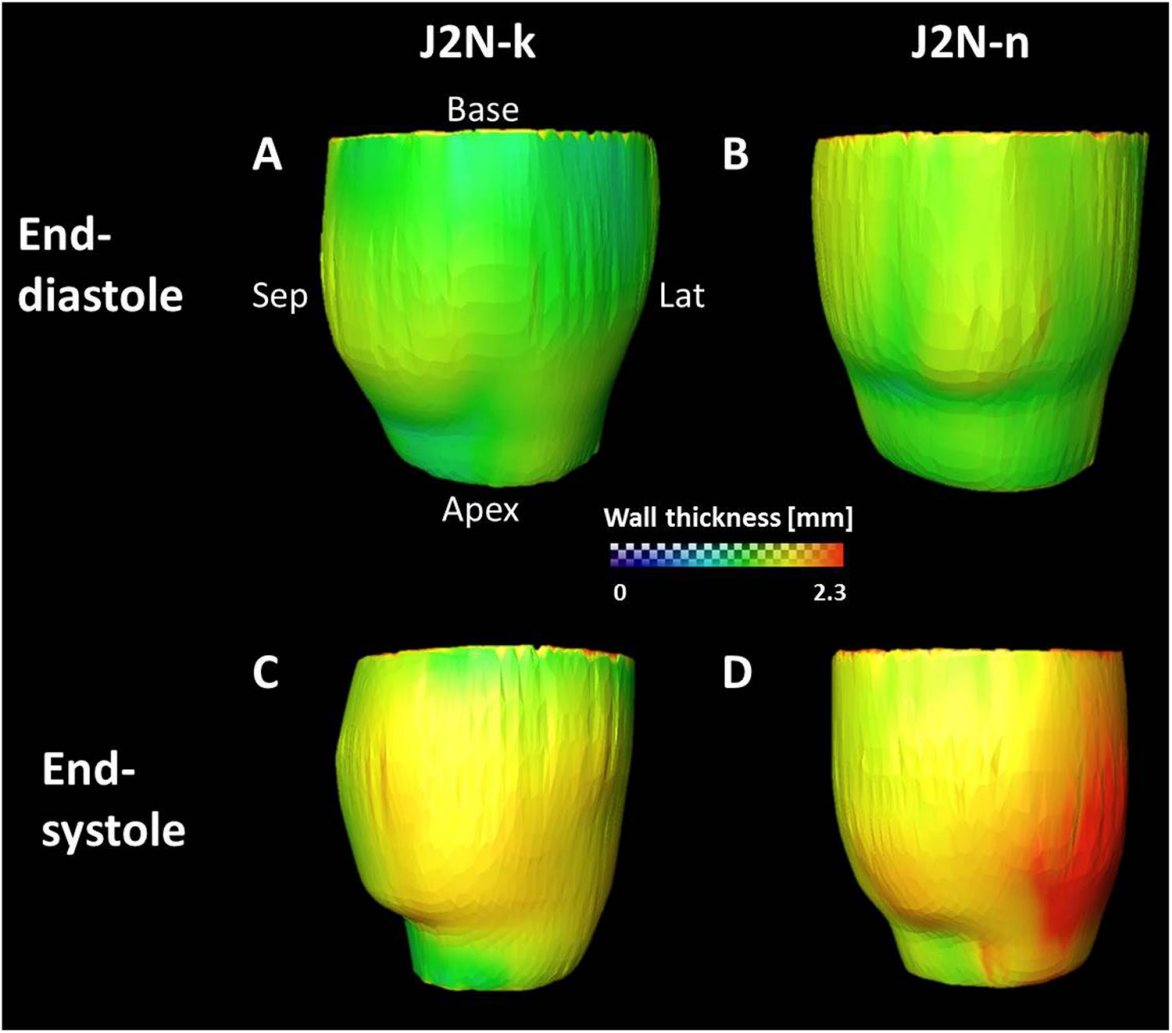
Typical three-dimensional (3D) color-coded mapping for the left ventricular (LV) wall thickness in J2N-k cardiomyopathic and J2N-n control hamsters. The colored bars represent the wall thickness from 0.0 mm to 2.3 mm. The 3D images show an anteroposterior (A → P) view at end-diastole (**A**) and end-systole (**C**) in J2N-k cardiomyopathic hamsters and J2N-n control hamsters (**B** and **D**).

3D color-coded mapping of the LV wall thickness at end-systole and end-diastole is shown in Fig. 3. The colored bar represents the wall thickness from 0.0 mm to 2.3 mm. 3D images obtained in the anteroposterior (A → P) view at end-diastole (Fig. 3A) and end-systole (Fig. 3C) for J2N-k cardiomyopathic hamsters are shown. 3D images obtained in the anteroposterior (A → P) view at end-diastole (Fig. 3B) and end-systole (Fig. 3D) for J2N-n control hamsters are also shown. At end-systole, the lateral wall of the left ventricle in J2N-k hamsters was thicker than that in J2N-n control hamsters. However, there were no differences observed between the two groups at end-diastole.

### LV wall thickness in J2N-k cardiomyopathic and J2N-n control hamsters

**Figure 4:**
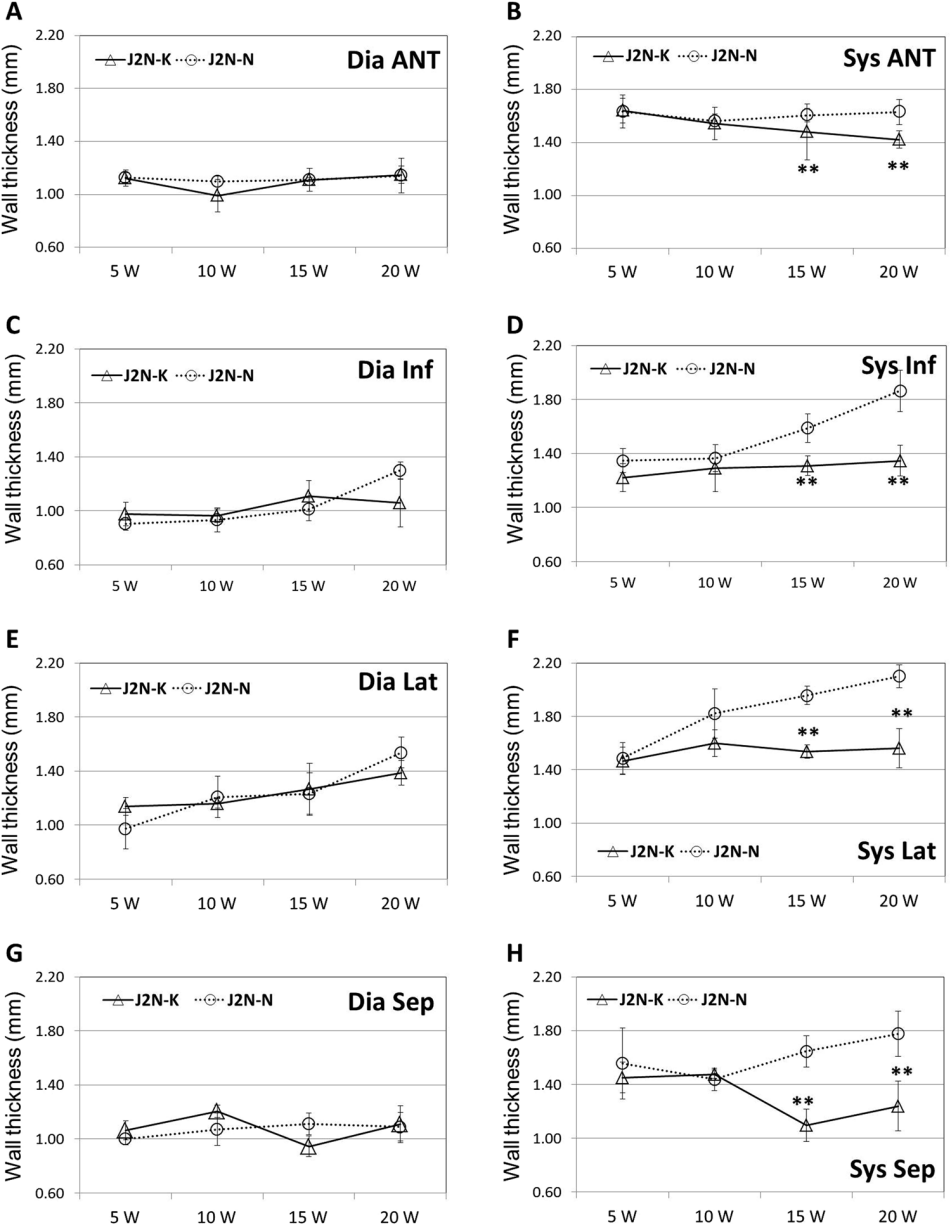
Left wall thickness in J2N-k cardiomyopathic and J2N-n control hamsters. The heart walls of J2N-k hamsters at 15 and 20 weeks are thicker than those of controls at end-diastole (Dia); however, they are not significantly different at end-systole (Sys). Anterior (Ant) wall, inferior (Inf) wall, septum (Sep), and lateral (Lat) wall. *p < 0.05. *p < 0.01.

As shown in Fig. 4A,C, E, and G, there were no differences between the two groups in the thickness of any wall at end-diastole. However, the thickness of the anterior wall (Fig. 4B), inferior wall (Fig. 4D), lateral wall (Fig. 4F), and septum (Fig. 4H) at end-systole was significantly lesser in J2N-k hamsters than in controls at 15 and 20 weeks of age (p < 0.01 for all walls at 20 weeks of age).

### Comparison of cine-MRI and echocardiography measurements

**Figure 5:**
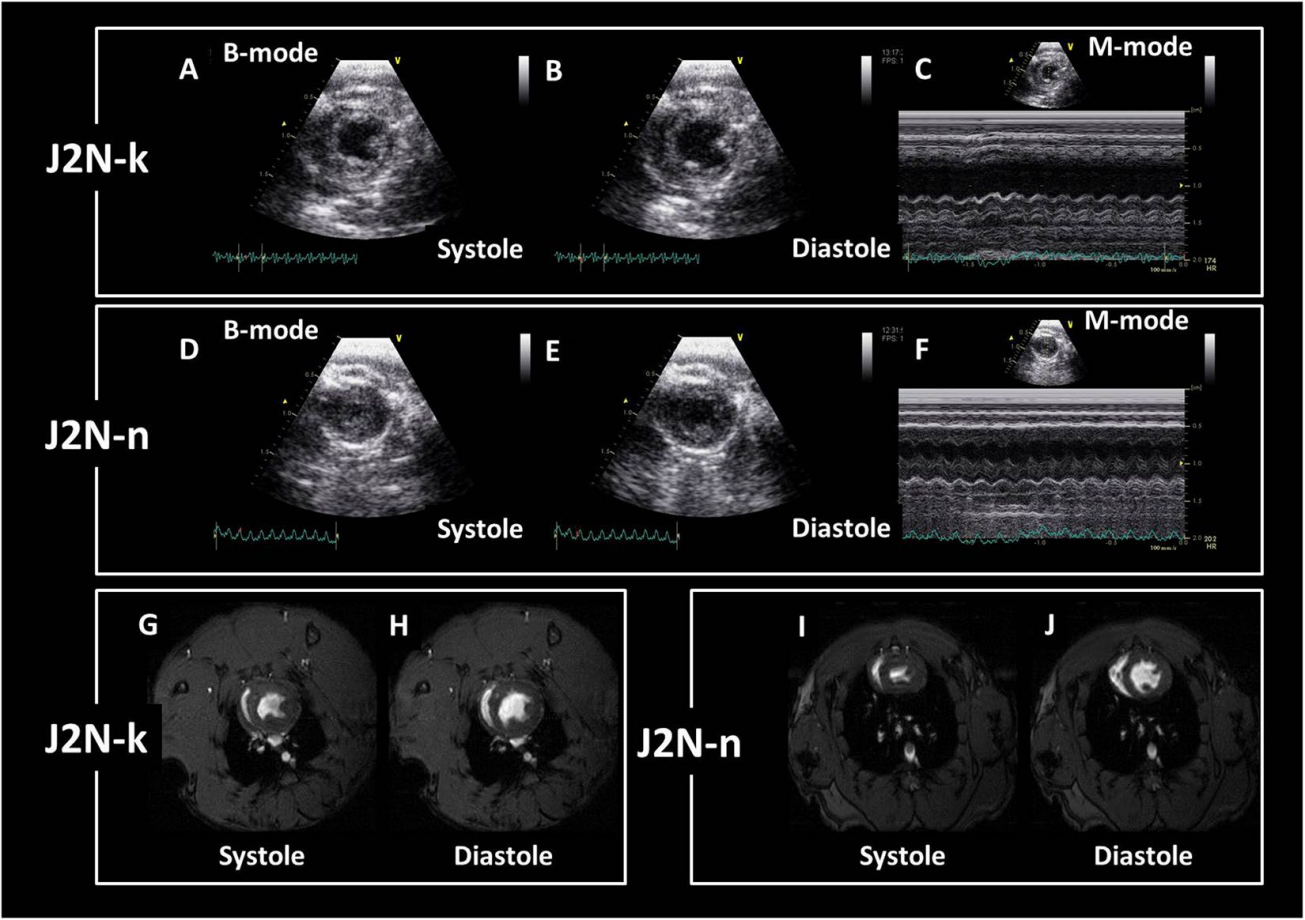
Examples of typical short-axis (SA) view images obtained using echocardiography and magnetic resonance imaging (MRI) for J2N-k cardiomyopathic and J2N-n control hamsters. (**A**) B-mode SA view images for J2N-k hamsters at end-systole. (**B**) B-mode SA view images for J2N-k hamsters at end-diastole. (**C**) M-mode SA view images for J2N-k hamsters. (**D**) B-mode SA view images for J2N-n hamsters at end-systole. (**E**) B-mode SA view images for J2N-n hamsters at end-diastole. (**F**) M-mode SA view images for J2N-n hamsters (**G**) SA view magnetic resonance images for J2N-k hamsters at end-systole. (**H**) SA view MR images for J2N-k hamsters at end-diastole. (**I**) SA view MR images for J2N-n hamsters at end-systole. (**J**) SA view MR images for J2N-n hamsters at end-diastole.

Typical cine MRI and echocardiography images for J2N-k cardiomyopathic and J2N-n control hamsters are shown in Fig. 5. Cine-MRI revealed a decrease in EF in J2N-k (46.5% ± 1.8%) hamsters compared with that in controls (78.1% ± 0.9%) at 20 weeks of age. Echocardiography also showed a decrease in EF in J2N-k (47.8% ± 0.9%) hamsters compared with that in controls (80.2% ± 5.4%) at 20 weeks of age. With regard to the wall thickness, MRI showed a decrease in the thickness of the inferior wall and septum in J2N-k hamsters compared with that in controls at 20 weeks of age, while echocardiography showed a decrease in the thickness of the inferior wall and septum in J2N-k hamsters compared with that in controls at 20 weeks of age (Table 2). There were no significant differences in EF and wall thickness values between MRI and echocardiography measurements. Cardiac movies obtained using echocardiography are shown in Sup.3 (J2N-k cardiomyopathic hamster, SA view) and Sup.4 (J2N-n control hamster, SA view). In addition, cardiac movies obtained using MRI are shown in Sup.5 (J2N-k cardiomyopathic hamster, SA view) and Sup.6 (J2N-n control hamster, SA view).

**Table 2:** Wall thickness of J2N-k cardiomyopathic and J2N-n hamsters measured by MRI and echocardiography.

	J2N-k		J2N-n	
	Diastole	Systole	Diastole	Systole
Inferior				
MRI	1.04 ± 0.16	1.43 ± 0.08	1.30 ± 0.06	1.86 ± 0.15
Echocardiography	1.05 ± 0.24	1.59 ± 0.23	1.32 ± 0.002	2.02 ± 0.06
Septum				
MRI	0.87 ± 0.08	1.10 ± 0.11	0.97 ± 0.04	1.80 ± 0.10
Echocardiography	0.79 ± 0.13	0.92 ± 0.06	0.92 ± 0.03	1.96 ± 0.05

Values are expressed as means ± standard deviation.

### Histology

**Figure 6:**
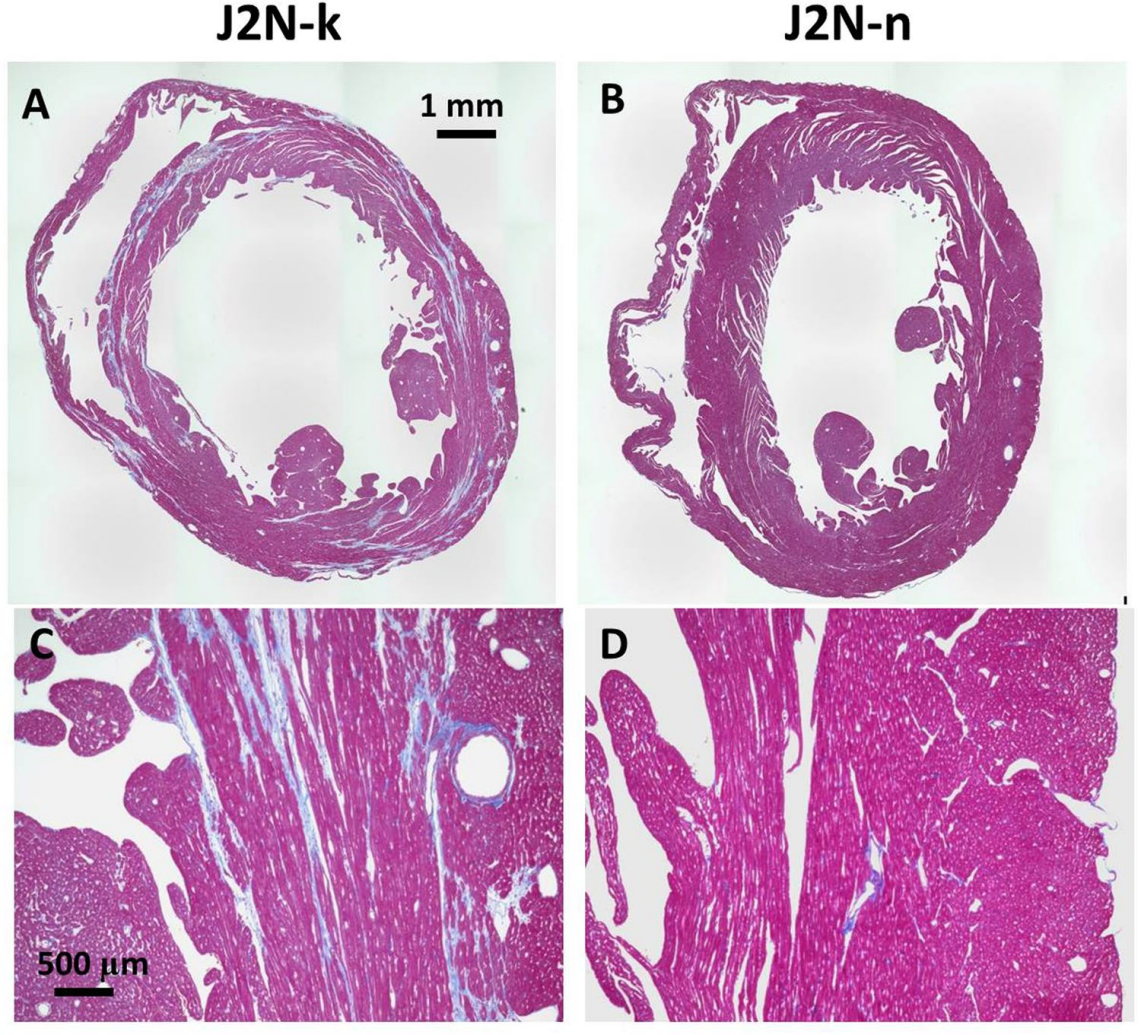
Histology of Masson’s trichrome (MT)-stained sections obtained from J2N-k and J2N-n hamsters. Dilatation of the left ventricular (LV) chamber is observed in 20-week-old J2N-k hamsters (**A**). MT staining depicts no fibrosis in the J2N-n control myocardium, whereas it shows interstitial fibrosis in the J2N-k cardiomyopathic myocardium (**A** and **D**).

Hamster hearts were treated with MT stain to demonstrate fibrosis (blue) at 20 weeks of age. MT staining indicated that fibrosis in J2N-k hamsters was significantly increased at 20 weeks of age (J2N-k: 14.7% ± 4.1%, J2N-n: 1.1% ± 0.4%, p < 0.001). MT-stained LV sections showed dilatation of the LV chamber in 20-week-old J2N-k hamsters. Considerable fibrosis, a decrease in the number of cardiomyocytes, and hypertrophic changes in the remaining cardiomyocytes were observed in J2N-k hamsters (Fig. 6A and C), while there were no significant changes in the hearts of J2N-n hamsters (Fig. 6B and D). Optical microscopy with MT staining depicted no fibrosis in the control myocardium and interstitial fibrosis in the J2N-k cardiomyopathic myocardium in the short-axis slice of the mid-left ventricle.

## Discussion

To the best of our knowledge, this is the first report to assess the LV function and wall thickness in J2N-k cardiomyopathic hamsters using serial 11.7-T MR cine imaging. EF and wall thickness evaluated by MRI were similar to those evaluated by echocardiography in both J2N-k and J2N-n hamsters. Our longitudinal study revealed impaired cardiac contractile function in cardiomyopathic hamster hearts. This was evident from the decrease in EF and CO and increase in ESV and EDV at 15 and 20 weeks of age in J2N-k hamsters. In addition, the present study presented 3D mapping of regional wall thicknesses at end-diastole and end-systole in cardiomyopathic and control hamsters. The heart walls of J2N-k hamsters at 15 and 20 weeks were thicker than those of control J2N-n hamsters at end-systole; however, there were no significant differences at end-diastole. Furthermore, interstitial fibrosis was observed in 20-week-old J2N-k cardiomyopathic myocardium using MT staining. These results indicated the presence of diastolic dysfunction in cardiomyopathic hamsters.

In a previous study, EF in 24-week-old cardiomyopathic hamsters was 35%–45% when evaluated by echocardiography, which is the gold standard tool for diagnosing cardiac disease^13^. In the present study, EF in 20-week-old cardiomyopathic hamsters was 47.8% ± 0.9% (echocardiography) and 46.5% ± 1.8% (MRI). These values are similar to those reported in previous echocardiography-based studies^14^. Moreover, the thickness of the inferior wall and septum in J2N-k hamsters showed a significant decrease compared with that in controls at 20 weeks of age in both our MRI and echocardiography studies. Therefore, the MRI-based measurements of EF and the wall thickness were similar to the echocardiography-based measurements in our study. In addition, the standard deviation values for both measurements were similar. This suggests that both our MRI and echocardiography methods are highly reproducible. Therefore, the present self-gated MRI study based on serial 11.7-T MR cine imaging could assess LV function and the wall thickness in the cardiomyopathic hamster model using subsequent long-term measurements.

The δ-sarcoglycan forms the SG complex with α-, β-, and γ-sarcoglycans, and the SG complex is speculated to play an important role in the function of dystrophin and the stability of sarcoplasmic reticulum membranes. A defect in the δ-sarcoglycan gene induces loss of other SG proteins in the heart and skeletal muscles^15^. In humans, a mutation in the dystrophin or other SG genes results in DCM^16^. The J2N-k cardiomyopathic hamster exhibits a deletion in the δ-sarcoglycan gene expressed in striated muscles^1^. J2N-k hamsters begin to show myocardial necrosis at around 5 weeks of age; they exhibit cardiac dilatation and dysfunction at approximately 20 weeks and finally die of congestive heart failure^3^. Other researchers have reported a considerable amount of fibrotic changes even in 8-week-old J2N-k hamsters using histopathological analysis with MT staining^17^. Moreover, J2N-k hamsters begin to show slight fibrosis at 8 weeks and exhibit moderate cardiac dysfunction, degeneration of cardiomyocytes, and interstitial fibrosis at approximately 20 weeks^2^. Optical microscopy for MT-stained sections in the present study depicted no fibrosis in the J2N-n control myocardium and only interstitial fibrosis in the J2N-k cardiomyopathic myocardium at 20 weeks. The accumulation of tissue fibrosis leads to a progressive decline in regional ventricular function in older J2N-k hamsters. Consequently, 10-week-old J2N-k hamsters had regional ventricular function similar to that of controls in the present study, while 15- and 20-week-old J2N-k hamsters exhibited significantly decreased wall motion. Another report showed that mild edema could be observed in hearts with DCM, and that the cardiomyocyte protein concentrations decreased by 9.5% in 16-week-old J2N-k hamsters compared with that in age-matched J2N-n hamsters^18^. In the present study, alterations in cardiac function and the wall thickness were not observed by MRI in 5- and 10-week old hamsters. However, a significant decrease in EF and CO and an increase in ESV and EDV were observed in J2N-k hamsters compared with those in controls at 15 and 20 weeks of age. Our results are consistent with those of previous reports describing that cardiac dilatation and dysfunction begin at 20 weeks of age in J2N-k hamsters^3, 18^.

An *in-vivo* imaging study using M-mode echocardiography revealed significant dilatation of the left ventricle and a decrease in LV contractility in J2N-k hamsters at 20 weeks compared with those in age-matched control J2N-n hamsters^17^. Moreover, age-dependent myocardial metabolic impairment was successfully imaged and quantitatively analyzed in J2N-k cardiomyopathic hamsters using fluorescent X-ray computed tomography (FXCT)^19^. Radio-iodinated BMIPP (^123^I-BMIPP) is a potential tracer for detecting myocardial fatty acid metabolism and ATP levels using single-photon emission computed tomography (SPECT)^20^. In a previous study, FXCT revealed homogeneous myocardial non-radio-iodinated BMIPP (^127^I-BMIPP) accumulation in normal hamsters from 8 to 25 weeks and cardiomyopathic hamsters aged 8 weeks^19^. However, BMIPP accumulated heterogeneously, and its uptake decreased in cardiomyopathic hamsters aged over 12 weeks^19^. These results show that the heart wall function of J2N-k hamsters decreased after 12 weeks of age. In our study, the thickness of muscles forming the heart walls in 15- and 20-week-old J2N-k hamsters showed a decreased at end-systole compared with that in J2N-n controls. Our results suggest that changes in regional wall motion can occur after 15 weeks in J2N-k hamsters, consistent with the accumulation of BMIPP observed with the use of FXCT^19^. Cardiac MRI is more sensitive to progressive alterations in cardiac function compared with other *in-vivo* imaging techniques^21, 22^. However, there are no reports on serial *in-vivo* imaging of disease progression in the same hamster over time. High-field 11.7-T MRI is capable of observing heart function with a high in-plane resolution of <200 µm and whole heart volumes and performing multidirectional assessments. Moreover, the self-gated navigator techniques used in our study showed a high signal-to-noise ratio and contrast ratios in small animal hearts, similar to non-self-gated MRI techniques^8–11^. Therefore, self-gated cine imaging using 11.7-T MRI can be used for simple cardiac function measurements in J2N-k and control J2N-n hearts. In the present study, we showed that it can be used to follow the development of cardiac decline in an animal model of DCM.

This study has a few limitations. Because of the relatively small number of model animals, individual variability within the same group of control or disease models should be taken into consideration. Larger numbers of model animals may reflect differences between J2N-k and control J2N-n hamsters in the early stages of disease progression. In addition, this study was conducted using a cardiomyopathic hamster model; therefore, the relevance of our findings with respect to changes in cardiac function in humans, particularly the relevant timing of the development of dysfunction, needs to be established. However, cardiac MR cine imaging can be performed for both humans and small animals; therefore, it is possible to make comparable measurements in human subjects and directly correlate them with the findings of this experimental protocol. Furthermore, elucidation of the time course of dysfunction in a hamster model is important for future studies seeking to evaluate the effectiveness of clinically relevant therapeutic strategies, such as peptide treatment^13^ and epicardial implantation of an atelocollagen^23^, for reversing this dysfunction in J2N-k cardiomyopathic hamsters at meaningful time points. Usually, the progression of myocardial interstitial fibrosis adversely affects both diastolic and systolic LV function^24^. However, this fibrotic change was reflected only in the systolic phase and was not detected in the diastolic phase in the present MRI study. Other advanced methods for the detection of myocardial changes need to be applied. Cardiovascular MRI, such as displacement encoding with stimulated echoes, tagged MRI sequences, and manganese-enhanced MRI, can assess advanced measures of cardiac mechanics and tissue alterations such as interstitial fibrosis, strain, and torsion^25–27^. We believe that advanced MRI techniques to identify early alterations in cardiac function in cardiac disease models, such as the J2N-k hamster, will prove useful for the assessment of disease development in the future.

In conclusion, cardiac imaging was used to examine hamsters using a high-field MR system with high temporal and spatial resolution in the present study. 3D mapping of the wall thickness allowed visualization of regional wall thicknesses at end-diastole and end-systole. The MRI-based measurements for EF and the wall thickness were similar to echocardiography-based measurements in 20 week-old J2N-k and J2N-n hamsters. Self-gated cine imaging based on ultra-high-field MRI can be used for accurate and easy measurement of cardiac function and the wall thickness in cardiomyopathic hamsters from 5 to 20 weeks of age.

## Methods

### Preparation of animal models

The Animal Welfare Committee of Osaka University approved this study. All experimental procedures involving animals and their care were carried out in accordance with the Osaka University Guidelines for Animal Experimentation and the National Institutes of Health Guide for the Care and Use of Laboratory Animals. Four-week-old male J2N-k (n = 6; weight, 65.3 ± 2.5 g; Japan SLC, Hamamatsu, Japan) and J2N-n control (n = 6; weight, 64.2 ± 3.2 g) hamsters were allowed to acclimatize at our facility for 1 week before the experiment. The animals had free access to food and water and were kept under standard laboratory conditions: 22–23 °C room temperature, approximately 50% humidity, and a 12/12-h light/dark cycle. Serial MR observations were performed at 5, 10, 15, and 20 weeks of age. In addition, cine MRI and echocardiography were performed at 20 weeks of age for three J2N-k and three J2N-n hamsters for comparison of cine-MRI and echocardiography measurements. In this comparison study, twenty-week-old male J2N-k (n = 3; weight, 117.0 ± 8.5 g; Japan SLC, Hamamatsu, Japan) and J2N-n control (n = 3; weight, 110.7 ± 2.1 g) hamsters were allowed to acclimatize at our facility for 1 week before the experiment.

### MRI

Serial MRI was conducted using an 11.7-T vertical-bore Bruker Avance II imaging system (Bruker Biospin, Ettlingen, Germany) and a volume radiofrequency coil for transmission and reception (m2m Imaging Corp., Cleveland, Ohio, USA). All MRI experiments were performed under general anesthesia using 1%–2% isoflurane (Abbott Laboratories, Abbott Park, IL, USA) administered via a mask covering the nose and mouth of the animals. Respiratory signals, body temperature, and heart rate were monitored using a physiological monitoring system (SA Instruments, Inc., Stony Brook, NY, USA). Body temperatures were continuously maintained at 36.0 ± 0.5 °C by circulating water through heating pads throughout all experiments. The center of the imaging slice was carefully positioned at the hamster hearts. First, a three-plane sequence was performed for the definition of slice orientation. Next, three established standard cardiac MRI views in hamsters (short axis, long axis four-chamber, and long axis two-chamber) were obtained using self-gated cine imaging with navigator echo. Finally, eight consecutive scans of the short axis from the apex to the base of hamster hearts were obtained in the long axis four-chamber and long axis two-chamber views. These 11 scans were used for fast low-angle shots with navigator echo (IntraGate, Bruker) using the following parameters: repetition time/echo time = 5.0/2.2 ms, flip angle = 10°, field of view = 4.0 × 4.0 cm, matrix = 256 × 256, slice thickness = 1.0 mm, number of repetitions = 300, eight concomitant slices covering the whole heart from the apex to base, 10 phases per cardiac cycle, expected heart rate = 400 beats per minute (bpm), expected respiratory rate = 30 bpm, in-plane resolution per pixel = 156 µm, acquisition time = 3 minutes 14 seconds per scan, total acquisition time = 35 minutes, and a total anesthesia time = approximately 1 hour.

### MRI data analysis

In short-axis images, end-diastolic and end-systolic frames were selected according to maximal and minimal ventricular volumes. The epicardial border was manually outlined and the LV cavity was segmented in both frames. The respective volumes were calculated as the area of each compartment multiplied by the slice thickness (1.0 mm). Based on end-systolic and end-diastolic volumes [ESV (µl) and EDV (µl), respectively), all parameters characterizing cardiac function, such as stroke volume [SV (µl) = EDV − ESV], ejection fraction [EF (%) = SV/EDV], and cardiac output [CO (µl/min) = HR (min^−1^) × SV], were calculated using OsiriX (v.5.8.1, Pixmeo, Bernex, Switzerland).

The hamster heart thickness was measured using the short-axis images. The area of hearts was divided into four areas: anterior wall, inferior wall, lateral wall, and septum. We mapped the average heart wall thickness, which was color-coded on a three-dimensional (3D) surface. The 3D color maps of the wall thickness were obtained by computing the distance along the vertex, normal to the normal’s intersection with the closest triangle in each vertex on the segmented heart wall surface using Amira ver 5.2 (Visage Imaging, Inc., San Diego, CA, USA) and techniques adapted from our previous report^28^. Next, the wall thickness was measured by region of interest (ROI) at end-diastole and end-systole in the four areas of the LV (around the fifth slice from the apex).

### Comparison of cine-MRI and echocardiography studies

Before echocardiography, MRI was performed for 20 weeks-old hamsters using the same previous MRI protocol. For echocardiography, anesthesia was induced with 3.0% isoflurane and a ventilation volume of 2.5 L/min and maintained with 2.0% isoflurane and a ventilation volume of 2.0 L/min. An anesthetic apparatus for small animals was used (SurgiVet, TK-5; Biomachinery, Chiba, Japan). The hamster was fixed in the supine position and the thoracic and inguinal regions were shaved. An electrocardiography device was attached for measurements and the heart rate was monitored. Transthoracic echocardiography was performed using Vivid 7 (10 MHz transducer, GE Healthcare, Horten, Norway). The frame rate was set at 235.4 frames/s. To evaluate the wall motion, an LV short-axis view at the papillary muscle level was obtained. With the M-mode method, the interventricular septal thickness, LV posterior wall thickness, and EF were measured offline using GE EchoPAC software (GE Healthcare, Horten, Norway).

### Histology

For the evaluation of fibrotic alterations in the heart tissues, all hearts were removed immediately after euthanasia and fixed in 4.0% formaldehyde neutral buffer solution. The heart tissues were dehydrated, embedded in paraffin, sectioned at a 2-μm thickness, and stained using Masson’s trichrome (MT) for histopathological analysis at the short-axis slice of the mid-left ventricle. The extent of cardiac fibrosis was assessed on MT-stained tissue specimens using microscopy (BZ-9000, Keyence, Osaka, Japan). After visualization with diaminobenzidine (Dako Japan Inc, Tokyo, Japan), tissue sections were briefly counterstained with hematoxylin. MT-stained cells for the assessment of fibrosis were identified in the following sequence. 1) The brightness and contrast were automatically optimized using Photoshop (v. 8.0.1, Adobe, Inc., CA, USA). 2) The blue-stained positive cells in MT-stained sections were segmented by the “Color threshold” plug-in of ImageJ (v. 1.40 g, National Institutes of Health, MA, USA). The threshold values of “Hue” ranged from 130 to 200. 3) The segmented images were divided by each color channel using the “Split channels” plug-in. 4) The blue-stained positive cells in the divided images were converted to white pixels using the “Invert” plug-in. 5) The ROI was set on the cardiac tissues in the converted images. 6) The percentage of the white pixels to cardiac tissues was calculated by the “Area fraction” plug-in. The “Area fraction” shows the percentage of pixels in the ROI that have been highlighted in white. This image analysis sequence was performed for all hamster heart sections.

### Statistical analysis

The estimated parameter values are expressed as means ± standard deviations. Differences in the estimated parameter values, including EF, EDV, ESV, CO, wall thickness, body weight, and heart rate, between groups were analyzed by two-way analysis of variance using Prism 5 (Version 5, GraphPad Software, CA, USA). We compared heart fibrosis evaluated by MT staining using unpaired *t*-tests. The statistical significance was determined by Bonferroni’s multiple comparison test. A p*-*value of < 0.05 was considered statistically significant.

## Electronic supplementary material

SUPPLEMENTARY INFO

Supplementary 1, ﻿Typical movie of a 4-chamber view obtained using magnetic resonance imaging for a J2N_k hamster

Supplementary 2,﻿ Typical movie of a 4-chamber view obtained using magnetic resonance imaging for a J2N_n hamster

Supplementary 3, T﻿ypical movie of an SA view obtained using b-mode echocardiography for a J2N_k hamster

Supplementary 4, Typical movie of an SA view obtained using b-mode echocardiography for a J2N_n hamster

Supplementary 5, Typical movie of an SA view obtained using magnetic resonance imaging for a J2N_k hamster

Supplementary 6, Typical movie of an SA view obtained using magnetic resonance imaging for a J2N_n hamster

## References

[1] Nakamura TY (2001). Stretch-activated cation channels in skeletal muscle myotubes from sarcoglycan-deficient hamsters. Am J Physiol Cell Physiol.

[2] Babick AP (2004). Cardiac contractile dysfunction in J2N-k cardiomyopathic hamsters is associated with impaired SR function and regulation. Am J Physiol Cell Physiol.

[3] Mitsuhashi S (2003). Defect of delta-sarcoglycan gene is responsible for development of dilated cardiomyopathy of a novel hamster strain, J2N-k: calcineurin/PP2B activity in the heart of J2N-k hamster. J Biochem.

[4] Ruff J (1998). Magnetic resonance microimaging for noninvasive quantification of myocardial function and mass in the mouse. Magn Reson Med.

[5] Slawson SE, Roman BB, Williams DS, Koretsky AP (1998). Cardiac MRI of the normal and hypertrophied mouse heart. Magn Reson Med.

[6] Wiesmann F (2002). Analysis of right ventricular function in healthy mice and a murine model of heart failure by *in vivo* MRI. Am J Physiol Heart Circ Physiol.

[7] Wiesmann F (2001). Dobutamine-stress magnetic resonance microimaging in mice: acute changes of cardiac geometry and function in normal and failing murine hearts. Circ Res.

[8] Fries P (2012). Comparison of retrospectively self-gated and prospectively triggered FLASH sequences for cine imaging of the aorta in mice at 9.4 Tesla. Invest Radiol.

[9] Fries P (2013). Comparison of self-gated and prospectively triggered fast low angle shot (FLASH) sequences for contrast-enhanced magnetic resonance imaging of the liver at 9.4 T in a rat model of colorectal cancer metastases. Invest Radiol.

[10] Verhaart IE (2012). Assessment of cardiac function in three mouse dystrophinopathies by magnetic resonance imaging. Neuromuscul Disord.

[11] Coolen BF (2011). Three-dimensional T1 mapping of the mouse heart using variable flip angle steady-state MR imaging. NMR Biomed.

[12] Zuo Z (2017). Assessment of Longitudinal Reproducibility of Mice LV Function Parameters at 11.7 T Derived from Self-Gated CINE MRI. Biomed Res Int.

[13] Mizuno Y (2015). Improvement of cardiac function after implanting the osteopontin-derived peptide SVVYGLR in a hamster model of dilated cardiomyopathy. Interact Cardiovasc Thorac Surg.

[14] Das S (2010). TNF-alpha-mediated signal transduction pathway is a major determinant of apoptosis in dilated cardiomyopathy. J Cell Mol Med.

[15] Sakamoto A, Abe M, Masaki T (1999). Delineation of genomic deletion in cardiomyopathic hamster. FEBS Lett.

[16] Melacini P (1999). Heart involvement in muscular dystrophies due to sarcoglycan gene mutations. Muscle Nerve.

[17] Takagi C (1999). Enhanced GRK5 expression in the hearts of cardiomyopathic hamsters, J2N-k. Biochem Biophys Res Commun.

[18] Maekawa K (2013). Global metabolomic analysis of heart tissue in a hamster model for dilated cardiomyopathy. J Mol Cell Cardiol.

[19] Takeda T (2008). Visualization of age-dependent myocardial metabolic impairment in cardiomyopathic model hamster obtained by fluorescent X-ray computed tomography using I-127 BMIPP. J Synchrotron Radiat.

[20] Fujibayashi Y (1990). Myocardial accumulation of iodinated beta-methyl-branched fatty acid analogue, iodine-125-15-(p-iodophenyl)-3-(R,S)methylpentadecanoic acid (BMIPP), in relation to ATP concentration. J Nucl Med.

[21] Bellenger NG (2002). Comparison of techniques for the measurement of left ventricular function following cardiac transplantation. J Cardiovasc Magn Reson.

[22] Stuckey DJ, Carr CA, Tyler DJ, Clarke K (2008). Cine-MRI versus two-dimensional echocardiography to measure *in vivo* left ventricular function in rat heart. NMR Biomed.

[23] Ishimaru K (2013). Synthetic prostacyclin agonist, ONO1301, enhances endogenous myocardial repair in a hamster model of dilated cardiomyopathy: a promising regenerative therapy for the failing heart. J Thorac Cardiovasc Surg.

[24] Kitamura M (2001). Collagen remodeling and cardiac dysfunction in patients with hypertrophic cardiomyopathy: the significance of type III and VI collagens. Clin Cardiol.

[25] Suever JD (2014). Simplified post processing of cine DENSE cardiovascular magnetic resonance for quantification of cardiac mechanics. J Cardiovasc Magn Reson.

[26] Kremer F (2013). 2-D strain assessment in the mouse through spatial compounding of myocardial velocity data: *in vivo* feasibility. Ultrasound Med Biol.

[27] Waghorn B (2008). Monitoring dynamic alterations in calcium homeostasis by T (1)-weighted and T (1)-mapping cardiac manganese-enhanced MRI in a murine myocardial infarction model. NMR Biomed.

[28] Saito S (2017). Mapping of left ventricle wall thickness in mice using 11.7-T magnetic resonance imaging. Magn Reson Imaging.

