# Supplementary material for: Longitudinal observations of progressive cardiac dysfunction in a cardiomyopathic animal model by self-gated cine imaging based on 11.7-T magnetic resonance imaging
Source: Sci Rep. 2017 Aug 22;7:9106. doi: 10.1038/s41598-017-09755-4 (PMC5567262; doi:10.1038/s41598-017-09755-4)
Supplement: Supplementary file 1 — SUPPLEMENTARY INFO [file 41598_2017_9755_MOESM1_ESM.pdf]

Longitudinal observations of progressive cardiac dysfunction in a cardiomyopathic animal model by self-gated cine imaging based on 11.7-T magnetic resonance imaging

Shigeyoshi Saito PhD [1,2*], Minori Tanoue BSc [1], Kasumi Masuda PhD [3], Yuki Mori PhD [2,4], Satoshi Nakatani MD, PhD [3], Yoshichika Yoshioka PhD [2,4] , Kenya Murase PhD [1,2]

[1] Department of Medical Physics and Engineering, Division of Health Sciences, Osaka University Graduate School of Medicine, Suita, Osaka, Japan, 560-0871

[2] Center for Information and Neural Networks (CiNet), National Institute of Information and Communications Technology, and Osaka University, Suita, Osaka, Japan, 560-0871

[3] Department of Functional Diagnostics, Division of Health Sciences, Osaka University Graduate School of Medicine, Suita, Osaka, Japan 560-0871

[4] Biofunctional Imaging Laboratory, Immunology Frontier Research Center (IFReC), Osaka University, Suita, Osaka, Japan, 560-0871

Supplemental files (6 files)

Supplementary 1

J2N_k_20weeks.avi

Typical movie of a 4-chamber view obtained using magnetic resonance imaging for a J2N_k hamster

Supplementary 2

J2N_n_20weeks.avi

Typical movie of a 4-chamber view obtained using magnetic resonance imaging for a J2N_n hamster

Supplementary 3

K_Mpeg.wmv

Typical movie of an SA view obtained using b-mode echocardiography for a J2N_k hamster

Supplementary 4

N_Mpeg.wmv

Typical movie of an SA view obtained using b-mode echocardiography for a J2N_n hamster

Supplementary 5

k_SA.avi

Typical movie of an SA view obtained using magnetic resonance imaging for a J2N_k hamster

Supplementary 6

n_SA.avi

Typical movie of an SA view obtained using magnetic resonance imaging for a J2N_n hamster
